# Supplementary figures and images for: Modelling transcription with explainable AI uncovers context-specific epigenetic gene regulation at promoters and gene bodies
Source: PLoS Genet. 2025 Oct 23;21(10):e1011908. doi: 10.1371/journal.pgen.1011908 (PMC12604806; doi:10.1371/journal.pgen.1011908)

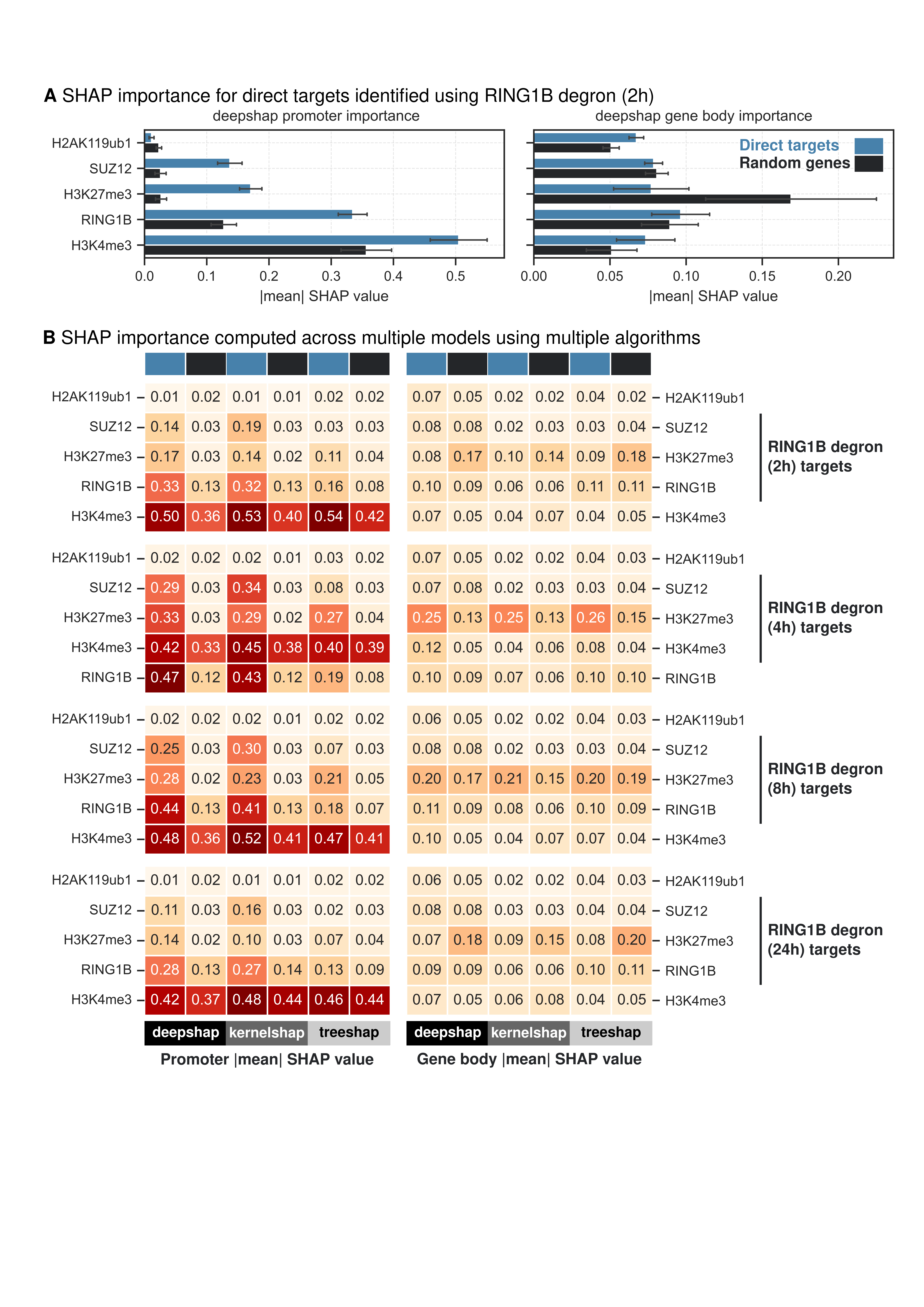

Supplement: S1 Fig — (A) Absolute mean DeepSHAP values computed for direct targets (RING1B degron 2h) and random genes at promoter and gene body contexts. Random subsampling is used to calculate intra-category variability and the error bars indicate the standard deviation of one-half. (B) Heatmap of absolute mean SHAP values for different algorithms DeepSHAP, KernelSHAP and TreeSHAP is plotted for direct targets (blue) and random genes (black) across all time points following RING1B degradation (2h, 4h, 8h and 24h). (TIFF) [file pgen.1011908.s001.tiff]

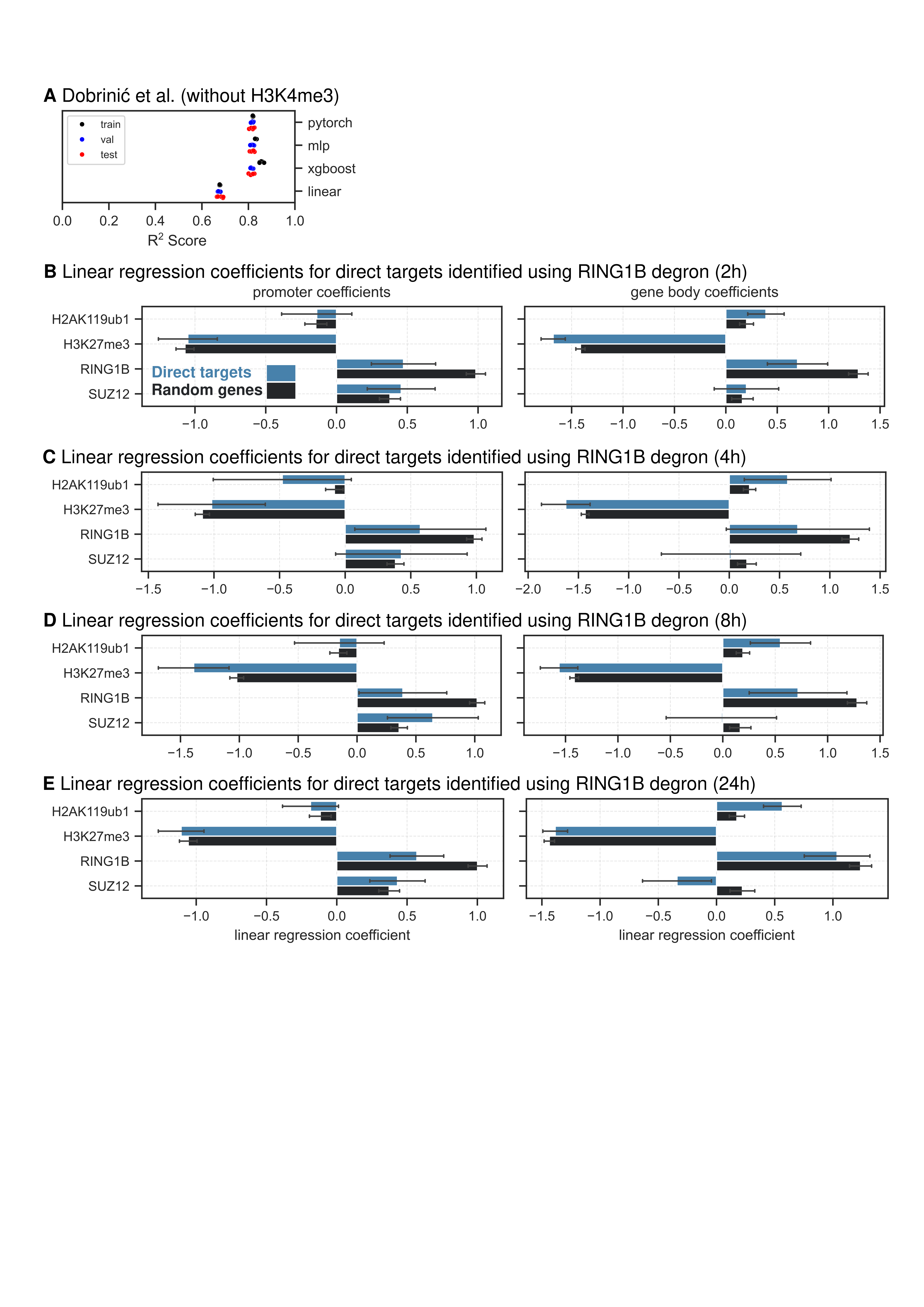

Supplement: S2 Fig — (A) Model performance for predicting RNA Pol-II occupancy based on chromatin-associated protein profiles for Dobrinić et al. without incorporating H3K4me3. Coefficients of determination (R2) are shown for training, validation and test datasets for 5 splits. Coefficients for chromatin-associated proteins and histone marks for direct targets (blue) and random genes (black) for (B) 2h (C) 4h (D) 8h and (E) 24h following RING1B degradation. (TIFF) [file pgen.1011908.s002.tiff]

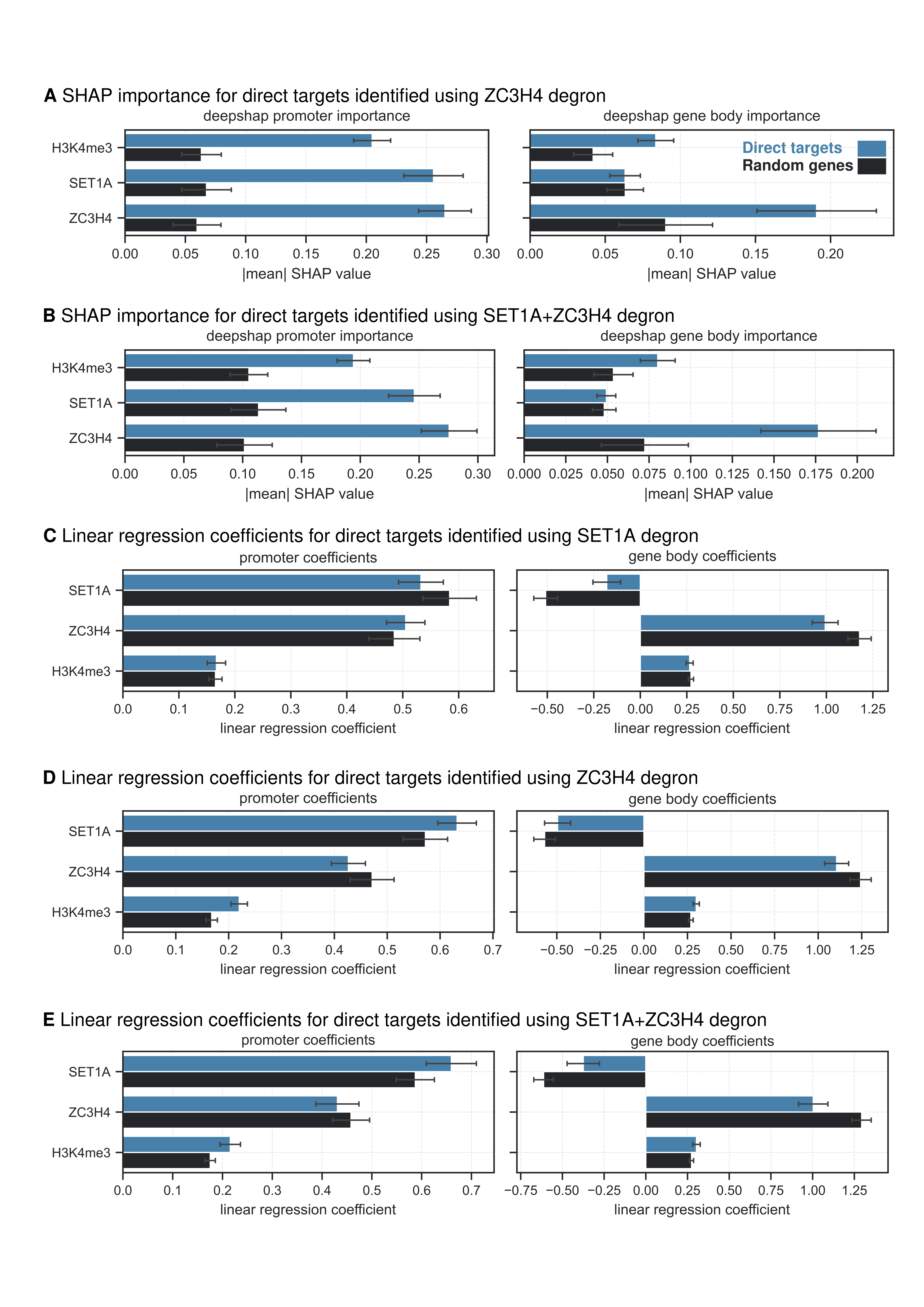

Supplement: S3 Fig — Comparison of absolute mean DeepSHAP values computed for (A) direct targets (ZC3H4 degron) and random genes, (B) direct targets (SET1A+ZC3H4 degron) at promoter and gene body contexts. Random subsampling is used to calculate intra-category variability and the error bars indicate the standard deviation of one-half. Coefficients for chromatin-associated proteins and histone marks for direct targets (blue) and random genes (black) for (C) SET1A degron (D) ZC3H4 degron and (E) SET1A+ZC3H4 degron. (TIFF) [file pgen.1011908.s003.tiff]

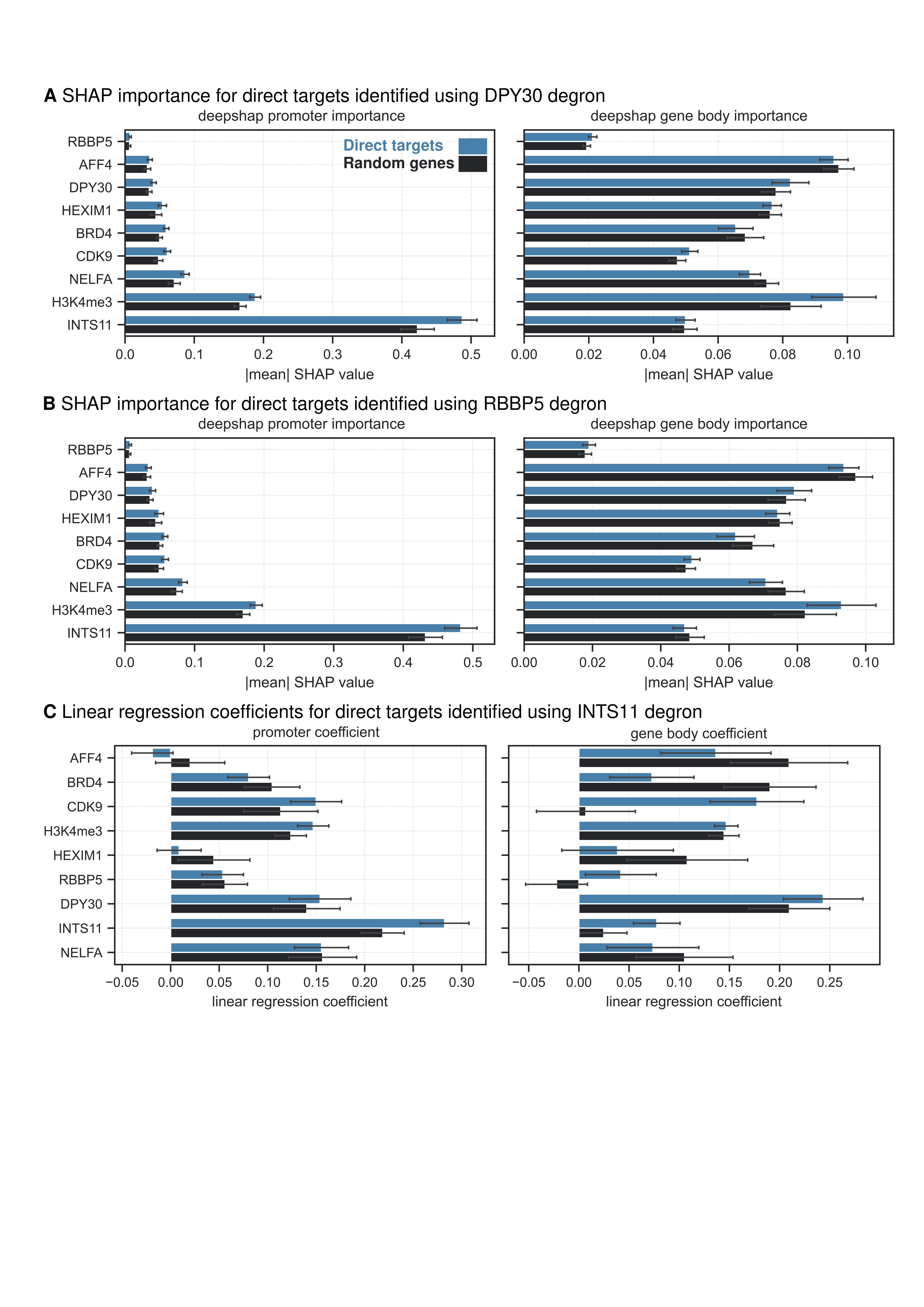

Supplement: S4 Fig — Comparison of absolute mean DeepSHAP values computed for (A) direct targets (DPY30 degron) and random genes, (B) direct targets (RBBP5 degron) at promoter and gene body contexts. Random subsampling is used to calculate intra-category variability and the error bars indicate the standard deviation of one-half. Coefficients for chromatin-associated proteins and histone marks for direct targets (blue) and random genes (black) for (C) INTS11 degron. (TIFF) [file pgen.1011908.s004.tiff]

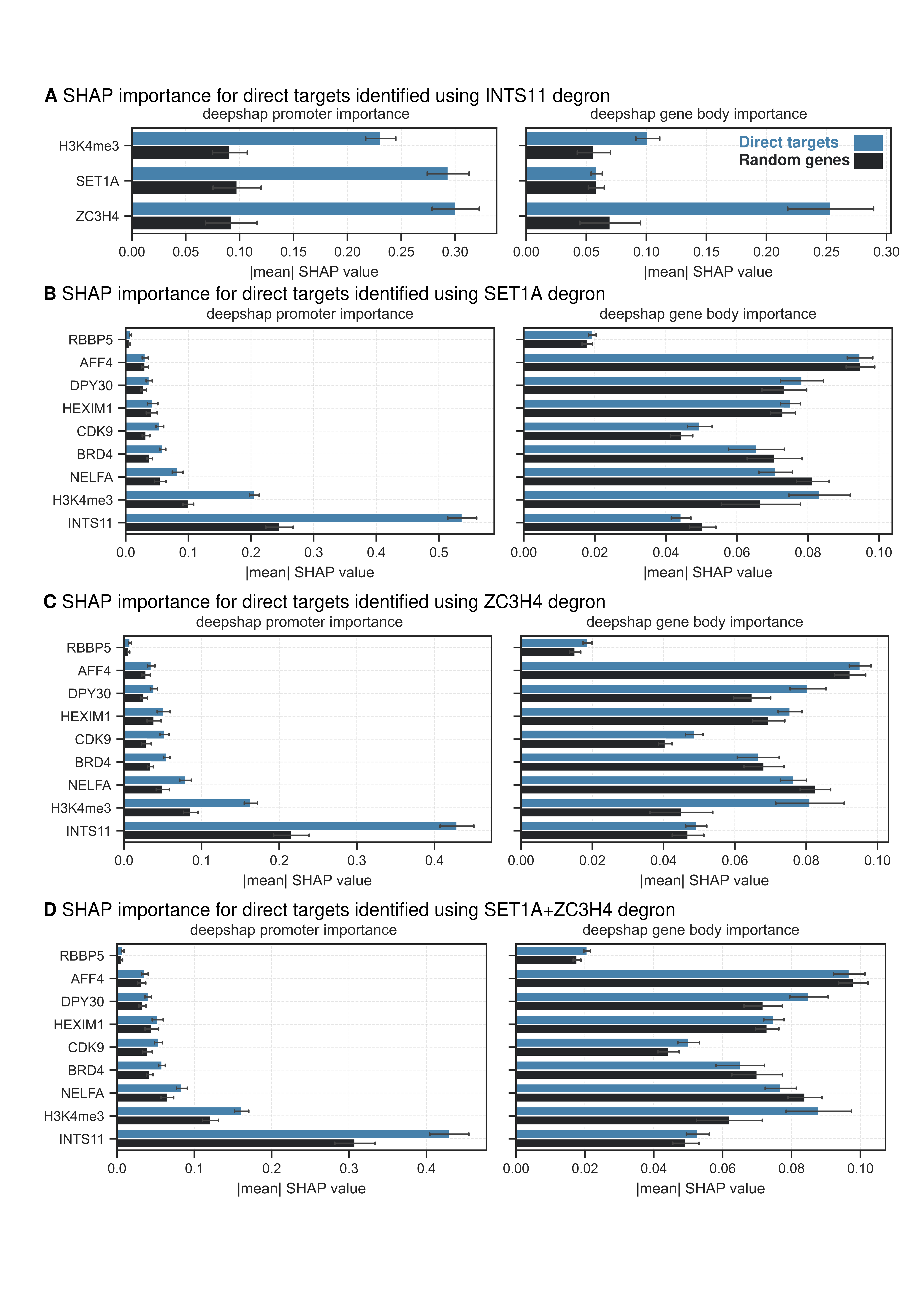

Supplement: S5 Fig — Random subsampling is used to calculate intra-category variability and the error bars indicate the standard deviation of one-half. (TIFF) [file pgen.1011908.s005.tiff]

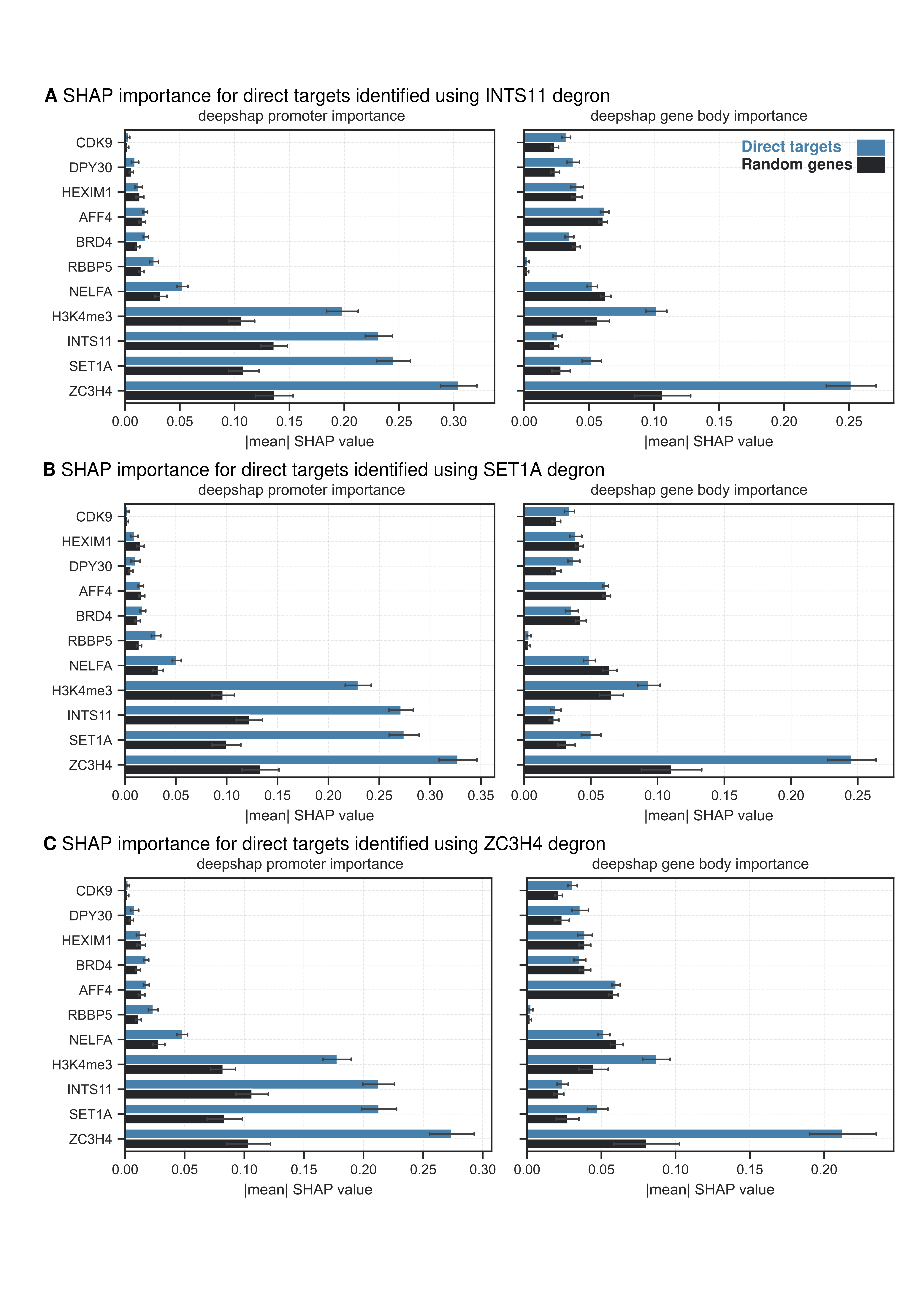

Supplement: S6 Fig — Absolute mean DeepSHAP values computed for (A) direct targets (INTS11 degron) (B) direct targets (SET1A degron) (C) direct targets (ZC3H4 degron) and random genes at promoter and gene body contexts. Random subsampling is used to calculate intra-category variability and the error bars indicate the standard deviation of one-half. (TIFF) [file pgen.1011908.s006.tiff]

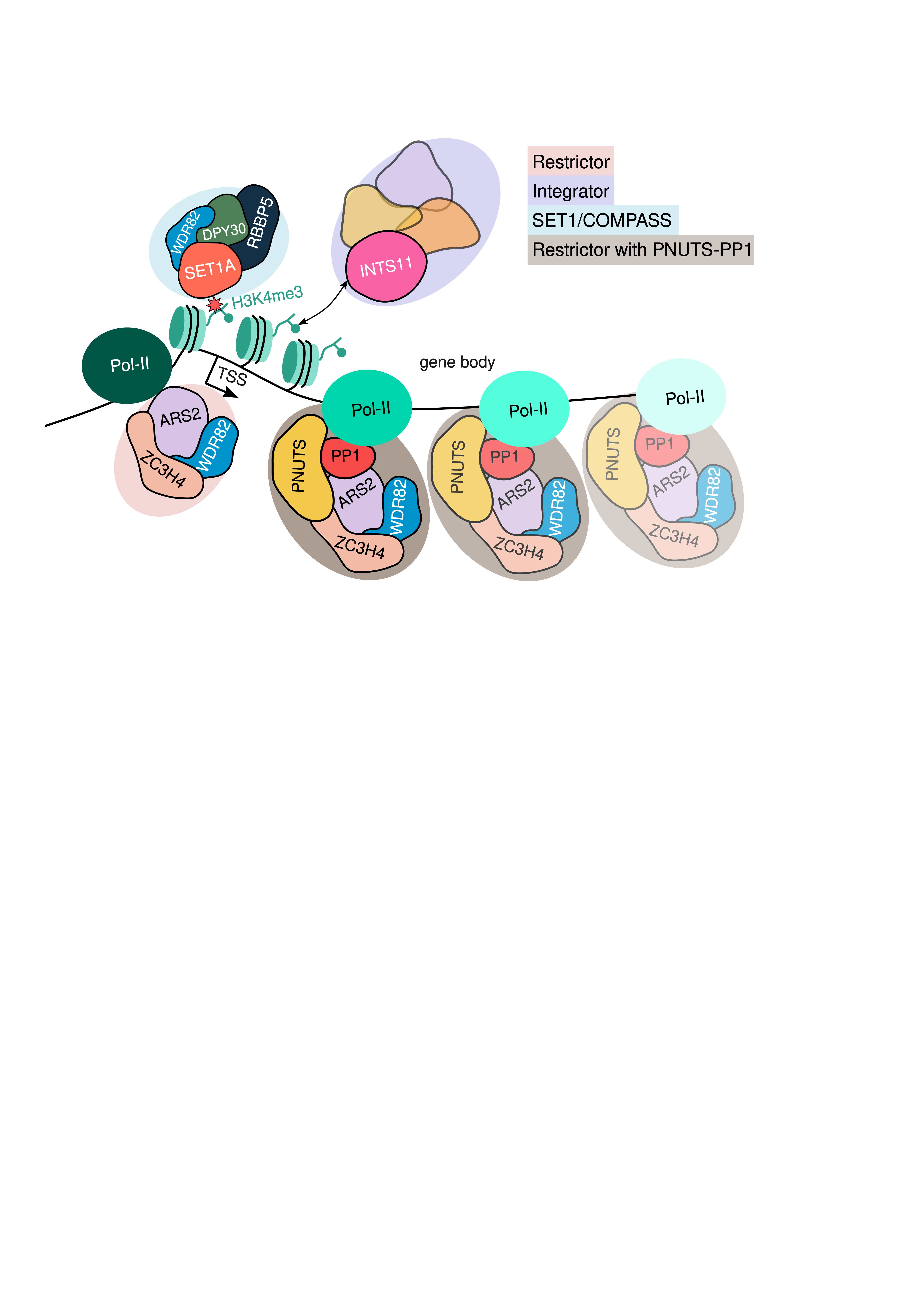

Supplement: S7 Fig — (TIFF) [file pgen.1011908.s007.tiff]
