# Supplementary material for: Modelling transcription with explainable AI uncovers context-specific epigenetic gene regulation at promoters and gene bodies
Source: PLoS Genet. 2025 Oct 23;21(10):e1011908. doi: 10.1371/journal.pgen.1011908 (PMC12604806; doi:10.1371/journal.pgen.1011908)
Supplement: Attachment — Submitted filename: reviewer_response.docx [file pgen.1011908.s009.docx]

Reviewer #1: Chhatbar et al present an analysis of transcriptional regulation based on ChIP-seq data using methods of explainable AI in combination with perturbation data. The approach consists of learning models of RNA polymerase occupancy at promoters as the prediction target using the binding patterns of chromatin associated factors as features. These models are then interpreted using Shapley additive explanations (SHAP-values). The authors make use of the gene specific feature importance quantified by the SHAP values to prioritize the key regulators of transcription. By comparing the SHAP values between direct targets of specific chromatin regulators and other genes in perturbation experiments the authors attempt to infer causal relationships. This aspect of the study is very noteworthy. There are however a number of issues that need to be be clarified.

*We thank the reviewer for their summary of our research and welcome their feedback on our noteworthy SHAP value comparison analysis.*

The manuscript lacks depth in the description of the methods. For example:

- how are promoter regions defined?
  - *The promoter regions are defined as +1kb and -1kb from the TSS (transcription start site) for every gene obtained from gencode annotation. This is visualised in Figure 1 and documented in Materials and Methods: ChIP-seq data analysis section (line numbers: 305-307).*
- how exactly was the Pol2 signal per gene quantified?
  - *Pol2 ChIP-seq, like every ChIP-seq signal in our study, was calculated in the following way. The promoter regions are defined as +1kb and -1kb from the TSS (transcription start site) and gene body was defined from +1kb to TES (transcription end site) for every gene obtained from gene annotation. After calculating the treatment and control pileup tracks using MACS2, fold enrichment tracks were calculated using bdgcmp from MACS2. To quantify the signal, bigWigAverageOverBed was used to calculate the mean signal over defined genomic regions, promoters and gene bodies. We apologise if this was not clear in our initial version and have updated the methods section with more detail about this. This is documented in Materials and Methods: ChIP-seq data analysis section (line numbers: 300-305) and S1 Appendix: Sequencing data preprocessing section.*
- Which gene annotations were used?
  - *Gene annotations were downloaded from the GENCODE project and the version M25 from this page* [*https://www.gencodegenes.org/mouse/releases.html*](https://www.gencodegenes.org/mouse/releases.html) *was used for the annotations.*
- What was the architecture of the MLP model?
  - *The architecture of the MLP model is different for different datasets. This information is present in Materials and Methods: Data preparation and modelling section (line numbers: 331-337).*
- Which hyper parameters were searched, which one were the optimal combination?
  - *We adopted the strategy of the best performing model where hyperparameter search for number of hidden layers, L2 regularisation term, activation function and maximum iterations was performed using Optuna. This information is present in Materials and Methods: Data preparation and modelling section (line numbers: 331-342) and S1 Appendix: Hyperparameter optimisation section contains the tables listing all the hyper parameters for all models.*
- how were the direct targets in the perturbation analyses defined?
  - *Direct targets were derived from TT-seq experiments performed after acute degradation of a chromatin-associated protein. DESeq2 was used to perform differential expression analysis between samples treated with dTAG/auxin and samples treated with DMSO (control). A gene is defined as a direct-target if p-adjusted value of that gene is less than 0.05 (p-adj<0.05). This information is described in line numbers 68-78 in detail.*

The manuscript lacks a comparison to other predictive modelling approaches (prediction of Pol2 from chromatin factors) that were carried out before. Notably, XGBoost has been used successfully and there is also a specific implementation of. SHAP for this algorithm. *We thank the reviewer for raising this important point. Our main aim is to learn a deep-learning model with sufficient accuracy and use SHAP values to infer relationships between chromatin-associated proteins and transcription. We hypothesise that the “model” is irrelevant and therefore we chose a generic MLPRegressor implementation from scikit-learn to allow for straightforward training and inference. As per the reviewer’s request, we update them with comparison of XGBoost Regressor and MLPRegressor alongwith SHAP interpretations in this updated manuscript. Our initial view was to keep the MLPRegressor because of its ease of implementation and fast training times. However, after this suggestion and additional work, we have trained multiple models: 1) A generic MLP Regression model in PyTorch 2) scikit-learn’s MLPRegressor 3) XGBoost Regression 4) Linear Regression. After training these models, appropriate SHAP algorithms are used to compute explanation values. These include: 1) DeepExplainer 2) KernelExplainer 3) TreeExplainer from the SHAP library ([https://shap.readthedocs.io/en/latest/api.html#explainers](https://shap.readthedocs.io/en/latest/api.html#explainers)) and linear regression co-efficients are used as a comparison. These additions are throughout all the sections and figures.*

The manuscript heavily relies on the interpretation of the model. Therefore it is key to evaluate the ability of the model to generalise. The authors should demonstrate the ability of the model to make predictions in cell lines not used for training. How is the prediction performance on other cell lines? *Thanks for raising this point. Epigenetic features, transcription factors and chromatin-associated proteins are highly tissue and cell-type specific. The degron experiments are performed in mESCs where the models are trained. We strongly believe that the direct targets can differ between different cell types and therefore comparing interpretations from one experiment in a particular cell type cannot be generalised with other cell types. Additionally, our regression models do not take DNA sequence into account which is the underlying common feature of different cell types. Therefore, our prediction was that these models are inherently non generalisable and they are so by design. Unfortunately, chromatin-associated proteins investigated in this study (especially INTS11, ZC3H4, SET1A/B) are not profiled in other cell lines. Nevertheless, we have tried to generalise a model learned in human HEPG2 cells (H3K27me3, H3K4me3, ZC3H4 and SUZ12 predicting Pol-II occupancy) and predicted the Pol-II occupancy in mouse ES cells. Our models were trained on data from HEPG2 cell line (source: ENCODE) and the withheld human genes in the test data set were converted to their orthologue mouse versions using Ensembl Biomart. Subsequently, X_test data from mESC cell line (source: ENCODE) was generated for the same features (H3K27me3, H3K4me3, ZC3H4 and SUZ12) and PolII ChIP-seq signal was predicted using weights learned from HEPG2 train and validation sets. Remarkably, in different species and different cell lines, our MLP models across all splits generalised with reasonable accuracy (~50%) while linear models showed no predictive power. These results provide proof of principle that if the same proteins are profiled in one cell line, they can be generalised to a different cell line. Due to the unavailability of data for the key features, we cannot incorporate this model in our study. We hope this attempt demonstrates generalisation.*

The advantage of using SHAP over other feature importance metrics should be demonstrated more clearly. For example: does it provide better results than using gini or similar metrics in random forest classifiers?

*SHAP values have many desirable qualities, especially theoretical consistency because of Shapley axioms, additivity and local explanations, which are not present in gini. Moreover,* [*Yasodhara et al. [arXiv:2110.00086]*](https://doi.org/10.48550/arXiv.2110.00086) *demonstrates that SHAP values are inherently more stable than gain feature importance (the regression version similar in spirit to gini). Nevertheless, as per the reviewer’s request in the above question, we have trained multiple models and quantified SHAP values using multiple explainers (including explainers compatible with XGBoost models e.g. TreeExplainer). We believe the additional work would help answer this question. These additions are throughout all the sections and figures. Additionally, there is a section in S1 Appendix: Interpretable machine learning techniques which attempts to explain the desirable properties of SHAP.*

The authors should also compare the their results to literature that uses graphical models (or partial correlations) to distinguish direct and indirect relations between chromatin regulators such as https://doi.org/10.1016/j.celrep.2016.01.008 or <https://doi.org/10.1093/nar/gku1234>

*We thank the reviewer for highlighting these relevant classic papers; we have now added this discussion item to clarify why they cannot be directly compared to in line numbers 273-279.*

Figure 4A is not easy to interpret. What do the grey lines represent?

*We apologise for this and have updated the figure with more details for easy interpretation. Because of the major revision and more additions, this figure is now the updated Fig6 and the figure caption is expanded to aid better interpretation.*

Reviewer #2: In "Unravelling epigenetic regulation of gene expression with explainable AI - a case study leveraging degron data," Chhatbar and colleagues demonstrate how to successfully predict RNA Pol-II occupancy based on binding data of various epigenomic factors.

The authors extensively use Shapley Additive Explanations (SHAP) values to interpret the biological underpinnings of their DNN-based predictions. Before publication, the authors should consider a couple of improvements and additional analyses.

1. Since this study primarily builds on the advantages of SHAP and because the readership of PLOS Genetics may not be familiar with Shapley values, I recommend including a paragraph explaining some of the fundamentals of SHAP theory. As the authors present a case study, such an introduction would be critical for all readers who want to conduct similar analyses. In particular, it would help to safeguard against problematic interpretations.

2. The interpretation of SHAP values can be problematic at times. This is particularly the case when the independence assumption is violated, for example, when using Lundberg and Lee. I understand that the marks investigated in the context of this study are, by design, dependent. While I acknowledge that this issue is inherent to the data, I want to encourage the authors to discuss this problem explicitly and explain how it should be addressed when interpreting the values. *We answer points 1. and 2. together and welcome these propositions from the reviewer. Now, we address this by utilising multiple SHAP algorithms and comment about it in line numbers 53-54 and we have added the paragraph explaining fundamentals of SHAP theory, the math behind it and interpretations in S1 Appendix: Interpretable machine learning techniques and brief summary in line numbers 79-82.*

3. There are some questions concerning the SHAP analysis. From the paper, the coalition sizes, i.e., the number of features, were somewhat limited. For instance, the analysis involving only GSE199805 used SET1A, ZC3H4, and H3K4me3 (M=3) to predict Pol-II occupancy. To what extent are these features correlated in the data? Is there a substantial difference between the correlations obtained from gene bodies and promoters? *We thank the reviewer for raising this point. We have added this information below and while these data are inherently correlated, our attempt is to use SHAP to gain insights on which feature is more important and therefore more likely to be responsible. Our SHAP value comparison between direct targets vs indirect targets addresses this but we make this more explicit in our S1 Appendix: Feature correlation section (page 6-7 of S1 Appendix). Relevant information is added in lines 357-361.*

4. It would be interesting to know how the overall Pol-II occupancy prediction performance changes for subsets of markers, e.g., when only using ZC3H4 and H3K4me3. Or even a single marker. *The aim is to find important marks for the direct targets from the “whole set” and therefore our initial strategy did not include comparisons of the prediction performance after removing certain marks. Because we want to find the importance of both ZC3H4 and H3K4me3, removal of either one would be counter intuitive. However, we appreciate this suggestion and therefore have implemented it in PRC1 models. To test this, we predicted Pol-II occupancy in our PRC1 models after removing H3K4me3. H3K4me3 is known to correlate with Pol-II occupancy and based on the reviewer’s suggestion, we assumed that the updated PRC1 model without H3K4me3 will have drastically lower predictive power than the old PRC1 model when H3K4me3 was included. Surprisingly, this was not the case and the updated PRC1 model still retained similar levels of accuracy with test R2 scores only dropping from ~85% to ~80%. We hope this answers the reviewer’s question. Relevant text is at lines 125-129 and model performance values can be found in Fig1B and S2 Fig A.*

5. To appreciate the feature importance differences for target and non-target genes, i.e., SHAP's potential to infer causality, it would be critical to know which strategies and thresholds were used to make this distinction: How were the target genes defined precisely? How many targets and non-targets are there for the individual analyses? *Thank you for raising this important point. Reviewer #1 has also asked this and we have answered the question there. This information is described in line numbers 68-78 in detail.*

6. I understand that the authors' GitHub repo probably contains all the information about the architecture, training procedure, thresholds, etc. Nevertheless, I recommend including essential information, such as the exact training strategy using "Scikit-learn's data-splitting functionality" (p. 11), e.g., to explicitly state how overfitting problems were addressed … *Thank you for addressing this important issue. The updated methods section contains all the relevant information about this. The update Materials and Methods section and S1 Appendix section contain this information.*

7. Since MLPs are rather bloated, it would be interesting to see how a decision tree-based approach, e.g., random forest or gradient boosting, would work. *We are thankful for the curiosity of the reviewer. As per the request, we have added multiple models and multiple explainers to address this question. This content is updated across the manuscript*

Minor issues:

- I may have missed it, but $|med(SHAP)|$ shown in several figures is not defined in the paper. *We apologise for this lack of information. The updated methods section reflects this, specially Materials and Methods: Prediction and interpretability analysis (line numbers: 352-356)*
- In Figure 1B, the test and train data points are not distinguishable. *We apologise for this. Our data points for re-trained models from different architectures and algorithms are replotted with smaller marker size to avoid this issue.*
